# The Impact of Obesity on Microglial Function: Immune, Metabolic and Endocrine Perspectives

**DOI:** 10.3390/cells10071584

**Published:** 2021-06-23

**Authors:** Vasileia Ismini Alexaki

**Affiliations:** Institute for Clinical Chemistry and Laboratory Medicine, University Clinic Carl Gustav Carus, TU Dresden, Fetscherstrasse 74, 01307 Dresden, Germany; VasileiaIsmini.Alexaki@uniklinikum-dresden.de; Tel.: +49-351-458-16273

**Keywords:** obesity, microglia, neuroinflammation, neurodegeneration, systemic inflammation, insulin resistance, dyslipidemia, cytokines, leptin, adiponectin, gut microbiome, glucocorticoids

## Abstract

Increased life expectancy in combination with modern life style and high prevalence of obesity are important risk factors for development of neurodegenerative diseases. Neuroinflammation is a feature of neurodegenerative diseases, and microglia, the innate immune cells of the brain, are central players in it. The present review discusses the effects of obesity, chronic peripheral inflammation and obesity-associated metabolic and endocrine perturbations, including insulin resistance, dyslipidemia and increased glucocorticoid levels, on microglial function.

## 1. Introduction

Obesity is a major health problem reaching worldwide pandemic proportions [1,2]. It is a result of the modern lifestyle, which is characterized by reduced physical activity and increased energy intake [1,2]. It constitutes an important health challenge as it substantially increases the risk for development of life-threatening conditions, such as type 2 diabetes mellitus, fatty liver disease, atherosclerosis, myocardial infarction and stroke [1,2]. Moreover, mounting evidence links obesity with enhanced cognitive decline during ageing and prevalence for development of neurodegenerative disease [3,4,5,6,7,8,9]. Obesity is associated with development of chronic inflammation in the adipose tissue [10], which gradually becomes systemic and may affect other organs, including the brain [8,11,12]. Microglia are innate immune cells of the central nervous system (CNS), which can respond to peripheral inflammatory signals and drive neuroinflammation [13,14,15,16]. The present review analyzes how obesity may promote microglia-mediated neuroinflammation and associated neurodegeneration through immune, metabolic and endocrine mechanisms. The roles of cytokines, adipokines, lipids, endotoxins and glucocorticoids are presented in this context. Special attention is given to neuroinflammation in the hypothalamus and hippocampus, due to the fundamental roles of these brain regions in whole body metabolism and cognition, respectively.

## 2. Microglia in Health and Neurodegenerative Disease

Microglia are resident immune cells of the CNS [15,17,18]. They derive from primitive macrophages of the yolk sac, are long-lived cells and under normal conditions their population is not replenished by peripheral immune cells but sustained through slow self-renewal [16,19,20,21,22]. Their cell population in the adult human and mouse brain is sustained by proliferation and apoptosis [22,23]. Microglia from different mouse brain regions display varying proliferative rates, with hippocampal microglia showing higher proliferation than their hypothalamic, cortical, cerebellar or midbrain counterparts [21,22]. Their signature was recently revealed with the help of single-cell approaches, which showed substantial differences between microglia and CNS-resident macrophage populations, as well as brain region- and age-dependent microglial heterogeneity [24,25,26,27,28]. In a healthy state, microglia display a unique profile, exemplified by high expression of homeostatic markers, such as Transmembrane Protein 119 (TMEM119), the puringergic receptor P2RY12, sialic acid binding Ig- like lectin H (SIGLECH) and probable G protein coupled receptor 34 (GPR34), which distinguishes them from other myeloid cells [24,29,30,31]. The adult microglial signature is gender-dependent, with female microglia presenting neuroprotective, while male microglia show rather inflammatory features [32].

Microglia have characteristic long processes, which they continuously extend and retract to scan their environment [33,34]. They orchestrate synapse pruning during development, monitor the function of synapses in adulthood and eliminate synaptic connections, the latter requiring ‘tagging’ of unwanted synapses by complement factors [35,36,37,38,39,40]. However, synapse elimination is also a hallmark of ageing and neurodegenerative disease, such as Alzheimer’s Disease (AD) [40,41,42]. Moreover, microglia provide trophic support to their neighboring cells by secreting neurotrophic factors, including nerve growth factor (NGF), brain-derived neurotrophic factor (BDNF), fibroblast growth factor (FGF) and Insulin-like Growth Factor (IGF), thereby regulating neuronal function and synapse formation [29,43,44]. In turn, NGF and BDNF can regulate microglial functions [13,14,45,46,47,48].

Upon acute injury, microglia direct their processes towards the lesion and trigger inflammatory responses in order to restrain the injury or infection [33,34,49,50,51]. They drive neuroinflammation by sustaining their own and astrocyte inflammatory activation [52,53]. For instance, TNF induces its own production, as well as the expression of IL-1β, IL-6 and iNOS in microglia [52]. Moreover, TNF, IL-1α and C1q derived from microglia promote inflammatory activation of a subset of astrocytes, termed A1 astrocytes [53]. A1 astrocytes have lost their ability to promote synapse formation or function, display compromised phagocytic capacity and exert neurotoxic effects [53]. In accordance, A1 astrocytes are abundant in brains of patients with AD, Huntington’s disease, Parkinson’s Disease (PD), amyotrophic lateral sclerosis (ALS) and multiple sclerosis (MS) [53].

Microglia are the main CNS phagocytes. They remove apoptotic cells and cell debris, which is necessary for resolution of inflammation and tissue repair [54,55]. They phagocytose free myelin, which is present in enhanced amounts in demyelinating disease or ageing [54,56,57]; myelin debris clearance is a prerequisite for remyelination and tissue repair [54,57,58,59]. In addition, they clear amyloid beta (Aβ) peptide; reduced Aβ clearance, commonly observed with advancing age, can sustain chronic microglia-mediated inflammation and drive neurodegeneration [54,60,61,62]. With advancing age, microglia display impaired phagocytic capacity and accumulate excessive amounts of myelin debris, which leads to cholesterol crystal formation, phagolysosomal membrane rupture and inflammasome activation [54,63,64,65,66]. Morphologically they undergo changes exemplified by deramification, amoeboid appearance, spheroid formation and fragmentation, designated as microglial dystrophy [67,68]. These microglia have reduced self-renewal capacity and can get dysfunctional or senescent [69,70,71]. Senescent microglia display increased expression of p16^Ink4a^ and are abundant in neurodegenerative pathologies, while their clearance prevents gliosis and neuropathology [70,71]. In addition, dystrophic or senescent microglia are less efficient in maintaining iron homeostasis and undergo ferroptosis [72,73]. Moreover, aged microglia may become immunologically ‘primed’, meaning they display increased expression of inflammatory factors and antigen presentation molecules and exhibit an exaggerated inflammatory response to stimuli [74].

In neurodegenerative disease, microglia lose their homeostatic profile and acquire a disease-associated signature (disease-associated microglia, DAM), characterized by enhanced expression of Apolipoprotein E (APOE), AXL, colony-stimulating factor 1 (CSF1), C-Type Lectin Domain Family 7a (CLEC7a), Integrin Subunit Alpha X (ITGAX), Cystatin F (CST7) and Basic Helix-Loop-Helix Family Member E40 (BHLHEe4) [29,75,76,77,78,79]. Moreover, in the context of neurodegenerative disease, microglial responses may be gender-dependent. For instance, in an AD mouse model female microglia progress faster than male microglia towards an activated state expressing major histocompatibility complex (MHC) type II and AD risk genes [80].

Summarizing, although neuroinflammation is a vital and protective response to injury, which is required for tissue regeneration, chronicity of neuroinflammation is destructive for neuronal and glial function and is a feature of neurodegenerative disease [81,82,83,84]. Although microglia might not be the initiator of neurodegenerative disease, their passage from a homeostatic to a DAM state, impaired phagocytosis and aberrant inflammation can promote disease development. To which extent microglia might be beneficial, destructive or insufficient to resolve tissue damage and promote regeneration in different neurodegenerative diseases remains unclear.

## 3. Microglia Are under the Influence of Peripheral Inflammation

The central and peripheral innate immune systems are in continuous communication [85]. Peripheral inflammation triggers a stress response by activating the hypothalamic-pituitary-adrenal gland (HPA) axis leading to enhanced production of glucocorticoids, in order to impede inflammation [85,86]. However, peripheral immune activation can also profoundly affect central immune function. Systemic inflammation induces an innate immune response in the brain, which is usually self-limited due to the activation of strict regulatory mechanisms restraining CNS inflammation [87]. Systemic administration of lipopolysaccharide (LPS), a component of the membrane of gram-negative bacteria, induces in the periphery strong production of pro-inflammatory cytokines, such as TNF and IL-6, which signal through or transpass the blood–brain barrier (BBB) and can target microglia [88,89,90,91,92,93]. LPS can also trigger cytokine release from BBB endothelial cells [94], while the penetration of LPS through the BBB is minimal [95]. However, a high dose of LPS enhances BBB permeability, especially in the frontal cortex, thalamus, pons-medulla and cerebellum, thereby augmenting the effects of peripheral pro-inflammatory signals in the brain [96,97]. In addition, LPS can also affect the brain function through stimulation of the vagal nerve. In turn, vagal nerve stimulation attenuates neuroinflammation and cognitive dysfunction induced by LPS and tilts microglia towards a neuroprotective phenotype potentially through the anti-inflammatory effects of acetylcholine or norepinephrine in microglia [98,99,100,101,102].

LPS strongly reduces microglial expression of homeostatic genes, such as TMEM119, SIGLECH, P2RY12 and GPR34, and phagocytosis-related genes, like Triggering receptor expressed on myeloid cells 2 (TREM2), MER Proto-Oncogene, Tyrosine Kinase (MERTK) and Transforming growth factor beta (TGFβ). In contrast, it triggers expression of inflammatory genes, such as IL-1β, TNF, IL-23p40, metabolic genes, such as inducible Nitric oxide synthase (iNOS), Phosphofructo-2-kinase/fructose-2,6-biphosphatase 3 (Pfkβ3) and Lactate dehydrogenase A (Ldhα), and anti-inflammatory genes, such as IL-10 [13,91,103]. In fact, distinct microglial activated profiles arise upon acute LPS-induced inflammation, as revealed through single-cell RNA sequencing [103]. Moreover, Wendeln et al. showed that microglia can be trained by a single low dose of peripherally administered LPS to respond more efficiently to a second stimulation with LPS. Microglial training involves epigenetic reprograming, in particular lysine 4 histone 3 methylation (H3K4me1), which persists even 6 months after stimulation. In contrast, three or more doses of LPS induce microglial tolerance. Microglial training promotes, while its tolerance alleviates, AD neuropathology in APP23 mice [91]. However, according to another study, repeated systemic LPS stimulation on four consecutive days maintains brain inflammation and induces loss of dopaminergic neurons in the substantia nigra through activation of the complement system [104].

Changes in microglial signature induced by peripheral inflammation can translate into alterations in neuronal and synaptic functions. For instance, postoperative cognitive dysfunction is associated with microglia-induced neuroinflammation triggered by operation-induced peripheral inflammation [105,106,107]. TNF, IL-6 and IL-1β generated by microglia can profoundly affect synaptic transmission and plasticity and underlie cognitive and behavioral alterations occurring in peripheral inflammatory disease [108,109,110,111,112]. Inflammation induced in the gut by intracolonic administration of 2,4,6-trinitrobenzene sulfonic acid (TNBS) significantly reduces synaptic transmission and plasticity in the hippocampus and increases pentylenetetrazole-induced seizure susceptibility, while these effects are reversed by TNF neutralization or minocycline-mediated inhibition of microglial activation [111,112].

Acute peripheral inflammation causes sickness behavior, primarily through the function of proinflammatory cytokines (IL-1β, TNF and IL-6) in the brain [113]. Long-term peripheral inflammation, such as in autoimmune diseases, systemic infections, cancer or immunotherapy, can lead to development of depression, especially in vulnerable patients or animals with preexisting sickness, due to decompensation of mechanisms regulating sickness behavior [113]. This is associated with HPA axis hyperactivity and glucocorticoid resistance, finally leading to uncontrolled inflammation [86,113]. Moreover, acute and chronic inflammation may aggravate existing neurodegenerative diseases [114,115,116,117,118]. Meta-analyses showed that levels of circulating pro-inflammatory cytokines (TNF, IL-1β and IL-6) are up-regulated in AD patients compared to age-matched control subjects and increased inflammation might even precede the outbreak of the disease [119,120]. Individuals with elevated pro-inflammatory cytokine levels over several decades are particularly prone to development of neurodegeneration [121,122]. Sustained presence of inflammatory signals may ‘prime’ microglia, which then exhibit an exaggerated inflammatory response when exposed to endogenous signals, such as Aβ or myelin [61]. Particularly in the aged brain, systemic inflammation can have detrimental effects [123,124,125]. Microglia from aged mice exhibit a stronger inflammatory response to peripheral LPS administration compared to microglia from young mice, which is associated with behavioral and cognitive disturbances [123,126,127,128,129,130]. Hence, chronic inflammatory diseases, such as rheumatoid arthritis, osteoarthritis, inflammatory bowel disease, chronic liver disease and type 2 diabetes mellitus, are often accompanied by fatigue, neuropsychiatric disorders such as depression, and cognitive disturbances [131,132,133,134,135,136].

## 4. Obesity-Associated Chronic Peripheral Inflammation

Obesity is one of the most common causes of chronic low-grade inflammation, which can lead to comorbidities such as type 2 diabetes mellitus and steatohepatitis, significantly impairing life quality [1,10,137]. Obesity-associated chronic inflammation in the adipose tissue and other organs, such as the liver, muscle and colon, is linked to metabolic disturbances, including insulin resistance and dyslipidemia, and is therefore termed ‘meta-inflammation’ [137,138,139,140,141]. The latter develops predominantly in subjects with increased visceral relative to subcutaneous adiposity [142,143,144]. In obesity, the adipose tissue undergoes structural, immune and metabolic reprograming [10]. It grows in size through adipocyte hypertrophy and hyperplasia, becomes hypoxic due to its inadequate vascularization and develops chronic inflammation due to secretion of inflammatory factors by adipocytes and immune cells [10,145,146]. Hypertrophic adipocytes secrete monocyte chemoattractant protein 1 (MCP1), TNF, IL-6, IL-1 and IL-8, triggering recruitment and inflammatory activation of immune cells [10,147]. Moreover, they secrete a number of pro-inflammatory adipokines, such as leptin, resistin and chemerin [10,147]. Leptin, the best-studied adipokine, regulates food intake and energy expenditure acting on the hypothalamus, while it also triggers pro-inflammatory responses in immune and endothelial cells [148,149]. Macrophages play a particularly important role in adipose tissue inflammation [10,150,151]. During obesity, macrophages accumulate in the adipose tissue and shift to a pro-inflammatory state, exemplified by increased expression of IL-1β, TNF and IL-6 [10,151,152,153]. In addition, neutrophils, cytotoxic CD8^+^ T cells and natural killer (NK) cells are recruited to the adipose tissue and promote adipose tissue inflammation [10,140,150,154]. Gradually, adipose tissue inflammation becomes systemic, affecting distant organs, such as the liver and muscle [10,11,137,155,156].

Chronic inflammation is associated with development of insulin resistance [157,158]. Mechanistically, IL-6 and TNF activate c-Jun N-terminal kinase (JNK) and nuclear factor ‘kappa-light-chain-enhancer’ of activated B-cells (NF-κB), which can block insulin signaling [158,159,160,161]. Consequently, obese subjects display insulin resistance and hyperinsulinemia and are therefore predisposed for development of type 2 diabetes mellitus [156]. Chronic inflammation and insulin resistance significantly increase the risk for development of various debilitating conditions, including non-alcoholic steatohepatitis (NASH), cardiovascular disease and neurodegeneration [10,137,162,163].

## 5. Obesity and Risk of Neurological Disease

Several studies have shown that obesity and high dietary fat intake are associated with increased risk of neuropsychiatric, cognitive and neurodegenerative disorders [9,164,165,166]. Meta-analysis studies showed that individuals with obesity or associated metabolic disorders in midlife have a significantly higher risk for development of dementia or AD later in life [164,167,168]. Moreover, numerous studies support a clear link between obesity and cognitive decline [165,169,170,171]. Obesity-associated comorbidities, including hypertension, type 2 diabetes mellitus and dyslipidemia, can further augment cognitive impairment [165]. Some studies also suggest that obesity in childhood and late adolescence may be a risk factor for MS [172,173,174,175]. In addition, epidemiological data strongly support an association between obesity and depression [166,176]. This association is stronger in women than men, can already exist in childhood and adolescence and is consistent across Western and non-Western countries [166,177]. Moreover, a large analysis showed that obese individuals with metabolic dysregulation are at greater risk for development of depression compared to obese subjects with a favorable metabolic profile [178].

**Figure 1:**
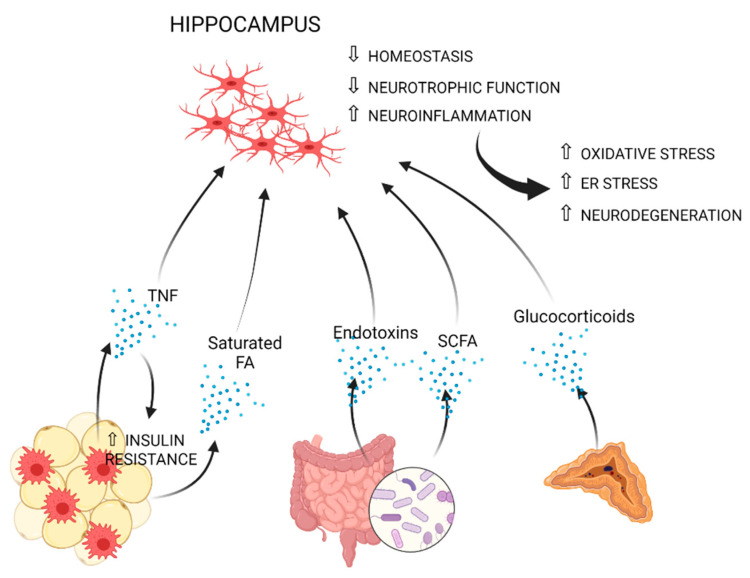
Peripheral immune, metabolic and endocrine routes, which may promote hippocampal microglial inflammation in obesity. Adipose-tissue-derived TNF and saturated fatty acids may exert microglial pro-inflammatory effects in the hippocampus. TNF promotes insulin resistance in the adipose tissue, which leads to enhanced lipolysis, further increasing the amount of saturated fatty acids in the circulation [10,88,89,140,188]. Gut microbiome-derived endotoxins and SCFA may also induce hippocampal neuroinflammation [189,190]. Chronically enhanced adrenocortical secretion of glucocorticoids may sustain microglia-mediated inflammation and neurodegeneration [191,192,193]. Collectively, these signals can lead to compromised homeostatic and neurotrophic function and sustained inflammatory activation of microglia, associated with oxidative stress, ER stress and neurodegeneration in the hippocampus [193,194,195,196,197,198,199,200,201,202,203,204].

**Figure 2:**
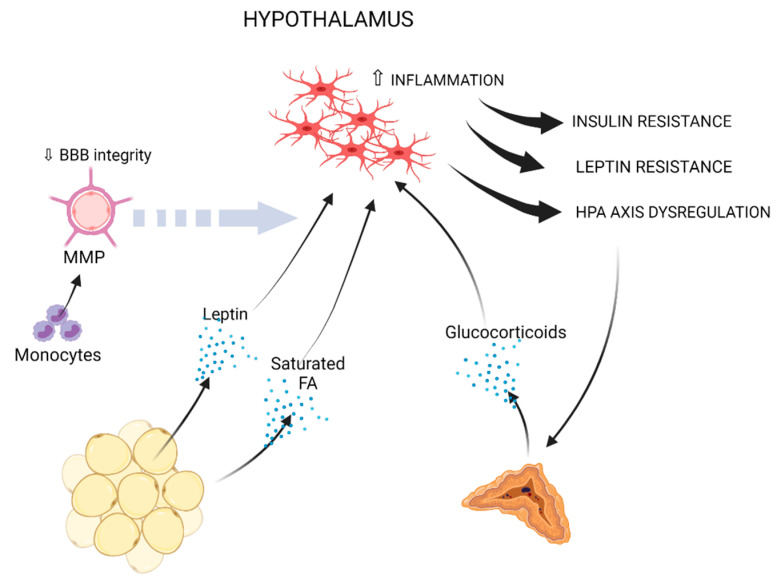
Obesity-induced peripheral immune, metabolic and endocrine factors, which may promote microglia-mediated inflammation in the hypothalamus. Fat-derived leptin and saturated fatty acids may promote neuroinflammation in the hypothalamus [212,256,257,259,260,264,266,267,268,269,277,278]. Circulating monocytes can drive BBB impairment through MMP production [269,279,280,281,282]. Hypothalamic neuroinflammation leads to insulin resistance, leptin resistance and HPA axis dysregulation [212,256,257,259,260,262,263,264,265,266,267,268,272,273]. Enhanced HPA axis activation drives increased glucocorticoid production by the adrenal gland, which may further sustain microglial inflammation [283,284].

Reduction of gray matter, compromised white matter integrity and lower brain volume, with the hippocampus and the prefrontal cortex being highly affected, are well-reported consequences of obesity or related metabolic perturbations [9,179,180,181,182]. Greater neuroinflammation and lower axonal density were also associated with obesity using diffusion basis spectrum imaging [183]. Microglial inflammatory activation is a common denominator of all aforementioned neurological conditions [61,184,185,186,187]. In the next paragraphs, mechanisms, which may causally link obesity to microglia-mediated inflammation, especially in the hippocampus and hypothalamus, are discussed (Figure 1 and Figure 2, and Table 1).

## 6. The Impact of Obesity on Microglia-Mediated Neuroinflammation

A large number of animal studies has shown that obesity profoundly alters brain plasticity and function. The hippocampus and hypothalamus are extensively studied in this context. High-fat diet (HFD) feeding of juvenile mice decreases neurogenic capacity and leads to defective neuronal connectivity in the dentate gyrus [211]. Obesity also leads to reduced hippocampus-dependent cognitive performance in adult mice and rats [194,195,231,232,233,234]. In accordance, obese mice display reduction in long-term potentiation, dendritic spines and sites of excitatory synapses in the hippocampus [194,195,235]. Diet-induced obesity promotes microglial inflammation, while astrocyte density or size are less affected [194,195,235]. Additionally, epididymal fat transplantation from leptin receptor mutant mice (*db/db*), which spontaneously develop obesity, into wild-type mice, causes hippocampal inflammation and disturbances in synaptic function and cognition [236]. Minocycline-mediated inhibition of microglial inflammation prevents dendritic spine loss and cognitive decline in obese mice, underscoring the critical role of microglia in obesity-induced cognitive impairment [195]. Moreover, early-stage obesity in rats interferes with prefrontal and perirhinal cortex-dependent cognitive function, accompanied by synapse and dendritic spine loss [237].

HFD feeding impairs microglial homeostasis in the hippocampus and amygdala. This is marked by increased expression of ionized calcium-binding adapter molecule 1 (IBA-1), CD11b, CD68 and MHCII, and decreased expression of homeostatic markers, including fractalkine and its receptor C-X3-C Motif Chemokine Receptor 1 (CX3CR1), TREM2 and its interactor DAP12 [194,195,196,197,238]. High-caloric diet also increases NOD-Like Receptor Protein 3 (NLRP3) expression, indicating inflammasome activation, and IL-1β production in hippocampus and amygdala-derived microglia [196,239]. In accordance, the IL-1 pathway was identified as an early sensor of metabolic changes triggered by overnutrition in adipose tissue macrophages [240]. Moreover, food intake alone triggers production of IL-1β by macrophages, which stimulates pancreatic insulin secretion and promotes inflammation [241]. Along these lines, intracerebral delivery of IL-1 receptor antagonist (IL-1ra) restrains hippocampal inflammation, synaptic dysfunction and cognitive impairment, indicating that IL-1β mediates obesity-associated deterioration of hippocampal function [236,239].

Intriguingly, in obese mice hippocampal microglia display increased synaptic inclusions, suggesting enhanced synaptic engulfment [195]. Partial knockdown of CX3CR1 or annexin V-treatment both attenuate obesity-associated cognitive decline by preventing microglial activation and phagocytosis, respectively [195]. These findings indicate that microglia drive obesity-associated dendritic spine loss and cognitive impairment through synaptic stripping in a CX3CR1-dependent manner [194,195]. However, a CX3CR1 antagonist reduces NMDA (N-methyl-D-aspartate) receptor subunit (NR2A), AMPA (α-amino-5-methyl-3-hydroxy-4-isoxazole propionate) receptor subunit GluR1, postsynaptic density protein 95 (PSD-95) and BDNF levels in the hippocampus [197]. In turn, impaired BDNF signaling may lead to decreased fractalkine and CX3CR1 expression in the hippocampus [197]. BDNF protein levels are also reduced in the brain cortex and synaptosomal fraction of obese mice, which is accompanied by increased TNF, IL-1β and oxidative stress and decreased mitochondrial respiration in the same regions [242].

Overnutrition leads to endoplasmatic reticulum (ER) stress in whole brain microglia and the hippocampus [198,240]. Strikingly, even a short (up to 10 days) period of HFD increases ER stress markers, apoptosis, Aβ and phosphorylated Tau protein levels in the hippocampus of mice [199]. Moreover, high caloric feeding upregulates genes of the heat shock protein (HSP) families HSP70, HSP90 and HSP40 in brain microglia [240]. In vitro, high glucose and palmitate up-regulate ER stress, apoptosis markers and inflammasome activation and down-regulate BDNF and synaptophysin expression in hippocampal cultures, while these effects can be reversed by activation of nuclear factor erythroid 2-related factor 2 (NRF2) [243].

The effects of HFD are exacerbated in aged animals. HFD feeding promotes stronger microglial inflammation and oxidative stress in the hippocampus of old compared to young mice [196,244,245]. In accordance, ageing augments obesity-induced deficits in hippocampal-dependent cognition, reduced synaptic density and impaired long-term potentiation [246]. Old HFD-fed mice also present increased IBA-1^+^ microglia numbers in the cerebellum, but not the cortex, compared to young HFD-fed mice [247]. Moreover, HFD feeding aggravates Aβ pathology, Tau phosphorylation, synaptic loss, microglia-mediated inflammation and cognitive deficits in AD mouse models [248,249,250,251]. For instance, western diet feeding of APP/PS1 mice increases the amount of TREM2^+^ microglia, which are implicated in AD pathology [245]. As recently shown using *App^NL-F/wt^* knock-in mice crossed with *ob/ob* mice, Aβ deposition shortens the lifespan of obese mice due to dysregulation of microglia and astrocytes [238]. In PD animal models, obese mice display greater dopamine neuron depletion in the substantia nigra and the striatum and increased neuroinflammation compared to lean controls [252,253]. Furthermore, obese animals display enhanced hippocampal neuroinflammation after induction of systemic inflammation [239,254,255]. Collectively, these data suggest that obesity may prime microglia, which upon activation by pro-inflammatory stimuli related to aging, neurodegenerative disease or systemic inflammation, trigger aberrant neuroinflammatory responses.

However, the first brain region affected by HFD feeding is the hypothalamus. In rodents, hypothalamic inflammation evidenced by increased expression of IL-1β, IL-6, suppressor of cytokine signaling (SOCS3), inhibitor of NF-κB kinase subunit b (IKBKB), F4/80 and Glial fibrillary acidic protein (GFAP) sets in a few days after HFD feeding start, hence prior to substantial weight gain or development of peripheral inflammation [256,257,258]. Infusion of glucose or oleic acid in the third ventricle triggers NF-κB activation in the hypothalamus independently of obesity [259]. Microgliosis, astrogliosis, neuronal injury and synapse loss are observed in the hypothalamic arcuate nucleus within the first week of HFD feeding [257,260,261]. These early responses subside temporarily, potentially through the activation of neuroprotective mechanisms [257]. However, during long periods of HFD feeding permanent hypothalamic neuroinflammation develops, featured by enhanced numbers of IBA-1^+^ microglia, astrogliosis, JNK and NF-κB activation, increased IL-1β, TNF and IL-6 expression, and induction of ER and oxidative stress [212,257,258,259,262,263,264,265,266]. The hypothalamic inflammatory response is driven by the accumulation and inflammatory activation of microglia in the mediobasal hypothalamus [256,267,268,269]. Additionally, expression of anti-inflammatory genes, such as IL-10 and CD206, is increased in the hypothalamus of mice with diet-induced obesity, perhaps in an effort to restrain or resolve inflammation [258]. Moreover, along with chronic inflammation, expression of homeostatic microglial markers, such as P2RY12, TMEM119, SELPLG, SLC2A5 and TREM2, is downregulated in hypothalamic microglia [258,269].

Prolonged inflammation in the hypothalamus instigates apoptosis of hypothalamic neurons and reduction of synaptic inputs, leading to impaired control of energy homeostasis [257,270,271]. Consequently, hypothalamic inflammation and injury result in leptin resistance and thus impaired control of food intake, thereby propelling obesity [263,272]. Moreover, hypothalamic inflammation promotes development of insulin resistance in the hypothalamus, further deteriorating whole body energy homeostasis [262,263]. Along these lines it was demonstrated that microglial depletion with a CSF1 receptor (CSF1R) inhibitor reduces food intake and body weight gain in HFD-fed mice [269]. Additionally, inhibition of microglial proliferation by intracerebral delivery of the antimitotic drug arabinofuranosyl cytidine dampens hypothalamic inflammation, restores leptin sensitivity and prevents body weight gain in mice [267].

Constitutive neuronal IKKβ/NF-κB activation impairs insulin and leptin signaling in the hypothalamus, consequently increasing food intake and body weight gain in HFD-fed mice, while overexpression of a dominant negative IKKβ exerts opposite effects [259]. Similarly, microglia-specific inhibition of NF-κB signaling in *CX3CR1^CreER^;Ikbkb^f/f^* mice reduces food intake and body weight gain upon HFD feeding [269]. Accordingly, mice with microglia-specific deficiency of the NF-κB negative regulator A20 display increased diet-induced hypothalamic microgliosis, enhanced body weight gain and perturbed whole organism energy expenditure [269]. The effects of hypothalamic IKKβ are mediated by SOCS3, a potent inhibitor of insulin and leptin signaling [259]. Moreover, JNK inhibition restores insulin signaling in the hypothalamus of obese animals and leads to reduced caloric intake and weight loss [262,263]. Finally, obesity-associated hypothalamic inflammation may also lead to HPA axis dysregulation and consequently stress disorders [273].

In concert with these findings, increased gliosis was demonstrated with magnetic resonance imaging (MRI) in the mediobasal hypothalamus of obese subjects [257,274,275]. IBA-1^+^ cells show aberrant morphology indicative of microglia dystrophy, characterized by enlarged cell bodies, shortened processes and cytorrhexis, in the hypothalamus of obese individuals compared to normal weight control subjects [258]. Finally, hypothalamic inflammation and damage detected with diffusion tensor imaging are associated with worse cognitive performance in obese individuals [276]. 

## 7. Mechanisms Mediating Obesity-Associated Neuroinflammation

### 7.1. The Impact of Obesity on Blood-Brain Barrier Function

A dense microcirculatory network ensures appropriate oxygen and nutrient distribution and removal of by-products from the neural tissue. Moreover, it forms the BBB and regulates entry of substances into the brain parenchyma. Hence, microvascular health is critical for brain homeostasis [165]. Cerebromicrovascular dysfunction or damage is an important cause of obesity-associated cognitive decline [165,285]. The mechanisms of obesity-associated endothelial disruption leading to cerebrovascular impairment are reviewed elsewhere [165].

The BBB is formed by endothelial cells of the microvessel wall, pericytes, the astrocytic endfeet, the inner vascular and the outer parenchymal basement membrane [286]. Microglial cells are in close connection with the BBB and together with the extracellular matrix they constitute the neurovascular unit [286]. Upon ischemia, microglia in the penumbra associate with blood vessels and engulf endothelial cells, which leads to BBB breakdown and enhanced entrance of blood serum components into the parenchyma [287]. In response to acute systemic inflammation, microglia accumulate around cerebral vessels before changes in BBB permeability are detectable, a contact that is protective for BBB integrity [288]. However, prolonged inflammation drives phagocytosis of the astrocytic endfeet and loss of BBB permeability [288]. Accordingly, LPS-activated microglia reduce the expression of tight junction proteins (zonula occludens-1-, claudin-5-, and occludin-like) through reactive oxygen species (ROS) production in an in vitro BBB model [289].

Several studies suggest that obesity may promote disruption of the BBB [165]. High-energy-diet consumption leads to reduced expression of tight junction proteins, particularly Claudin-5 and -12, in the choroid plexus and the BBB, and enhanced BBB permeability in the hippocampus [290,291]. Using a mouse model with tdTomato^+^ microglia and tdTomato^−^ peripheral myeloid cells, Valdearcos et al. showed that mice fed for 4 weeks a HFD display peripherally recruited myeloid cells in the hypothalamus [269]. The same study showed that C-C chemokine receptor type 2 (CCR2)^+^ monocytes appear in the mediobasal hypothalamus within the first week of HFD feeding [269]. The percentage of peripheral monocytes and neutrophils, identified as CD11b^+^CD45^high^ cells, is greater in western diet compared to control diet-fed animals in a CCR2-dependent manner [279]. According to the same study, CD11b^+^CD45^high^ but not CD11b^+^CD45^low^ cells, the latter representing microglia, display a proinflammatory signature, exemplified by high ITGAL, TREM1 and Osteopontin expression [279]. Diabetes mellitus and hypercholesterolemia also increase BBB permeability [292]. BBB integrity can be more strongly impaired in obesity in combination with ageing [244], while certain brain regions, such as the dorsal striatum and subregions of the hippocampus, may be more vulnerable than others [293]. In contrast, according to another study using bone marrow chimeric mice harboring actin-GFP bone marrow, peripheral myeloid cells are not present in the hypothalamus after 20 weeks of HFD feeding [258]. This discrepancy may be due to use of different mouse models, genetic backgrounds, experimental settings or diets in these studies.

Microglia depletion abolishes the recruitment of peripheral myeloid cells in the hypothalamus of HFD-fed mice, suggesting a critical role of microglia in the recruitment of circulating immune cells [269]. BBB disruption facilitates the entry of plasma constituents including IgG and fibrinogen into the brain parenchyma, which may promote microglial inflammatory activation [294,295]. Fibrinogen induces rapid and sustained responses of perivascular microglia through binding to CD11b leading to ROS release and axonal damage [295,296]. Moreover, obesity-induced BBB dysfunction is associated with upregulation of activating Fc-γ receptors, which can interact with IgG and promote microglia activation in the hippocampus of mice [244]. Activated microglia may cause further BBB impairment, thereby maintaining a vicious cycle of inflammation [280,289].

### 7.2. Circulating Monocytes

Obesity is associated with increased circulating monocyte numbers in humans and mice [297,298,299]. Mechanistically, the fat-derived alarmins S100A8/A9 locally stimulate macrophage Toll-like Receptor 4 (TLR4) signaling and thereby IL-1β production, which drives myelopoiesis in the bone marrow [297]. Additionally, in metabolically unhealthy obese individuals proinflammatory monocyte counts are increased compared to metabolically healthy obese control subjects [298]. In addition, monocytes acquire a more inflammatory profile in human obesity [300,301]. Hence, more pro-inflammatory monocytes may contribute to systemic and central inflammation in the obese state. Myeloid cells are important producers of matrix metalloproteinases (MMPs) [281,302]. Human macrophages express several MMPs, while murine macrophages predominantly produce MMP-12 and to a lesser extent MMP-9 [303]. Insulin and palmitate can increase MMP-9 expression in human monocytes [304,305]. In accordance, MMP-12 expression is up-regulated in the obese adipose tissue and supports nitric oxide synthesis in macrophages [306]. MMPs can promote BBB breakdown through degradation of tight junction and basal lamina proteins and thereby facilitate leukocyte infiltration [282]. Augmented MMP expression is a feature of neuroinflammatory and neurodegenerative disorders, including AD, PD and MS [307]. For instance, monocytes with enhanced MMP-1, -3, -7 and -9 mRNA expression are more frequent in MS compared to control patients [308]. Neutrophil-derived MMP-9 destructs type IV collagen, a key component of the BBB in *Mycobacterium tuberculosis* infection [309]. Leukemic cell-derived MMP-2 and -9 destroy tight junction proteins and thereby increase BBB permeability [310]. Taken together, MMPs secreted by circulating myeloid cells may additionally compromise BBB integrity in obesity.

### 7.3. Cytokines

In obesity, proinflammatory cytokines, such as TNF and IL-6, are produced predominantly by adipose tissue macrophages [10]. In accordance, TNF, IL-6 and IL-18 levels are elevated in the serum of obese compared to non-obese subjects [311,312,313], while IL-1β serum levels are increased in obese subjects with rheumatoid arthritis compared to non-obese control patients [314]. In addition, type 2 diabetes mellitus strongly correlates with increased serum levels of TNF and IL-6 [311,312]. TNF and IL-6 can affect the CNS either by signaling through their respective receptors on the BBB endothelium or by transpassing the BBB [88,89,90,315]. Cytokine uptake in the brain may be enhanced in metabolic disease due to reduced BBB integrity [290,291,292].

TNF skews microglia towards a proinflammatory and less phagocytic phenotype in vitro and in vivo [205]. Alone or in combination with IFNγ, it increases the expression of inflammatory genes, including C1R, IL-6, iNOS, apoptosis-inducing factor 1 (AIF1), CCR5, CD68, TLR4 and SOCS3, reduces expression of homeostatic genes, such as P2RY12, CX3CR1 and CSF1R, and decreases the expression of phagocytosis-related genes, such as TREM2 and TGFBR1 [92,206,316]. Moreover, it promotes its own production in microglia, thereby sustaining microglial inflammation [92,108]. TNF secreted by microglia mediates endothelial necroptosis, exacerbating BBB breakdown in ischemic stroke [317]. It also targets astrocytes, which then may exert toxic effects on neurons and oligodendrocytes [53]. In addition, TNF directly targets neuronal cells, increasing neuronal injury, CNS excitability and neural precursor apoptosis [111,318].

IL-6 has pleiotropic functions [319,320]. Neonatal overnutrition in rats leads to increased IL-6 serum levels and protein amounts in the hypothalamus and cerebellum, without concomitant changes in IL-6 mRNA levels, suggesting an increased uptake of circulating IL-6 [321]. Chronically elevated IL-6 levels in the CNS due to IL-6 overexpression in astrocytes in transgenic mice promotes microglial density, inflammation exemplified by increased IL-1β, TNF, IL-10, IL-6 and MHCII expression, and neurologic disease [322,323]. Moreover, IL-6-deficient mice are resilient to LPS-induced sickness behavior and do not develop age-associated baseline increase of IL-1β in the hippocampus [93]. However, IL-6-deficient mice develop disturbed carbohydrate and lipid metabolism, leptin resistance and mature-onset obesity, which can be reversed by intracerebroventricular or intrahypothalamic application of IL-6, suggesting an important role of IL-6 in the hypothalamic control of energy intake and metabolism [324,325]. Moreover, exercise-induced increase in IL-6 levels suppresses hyperphagia and hypothalamic inflammation [326] and counteracts the neurotoxic effects of TNF [327]. Finally, IL-6 mediates protective effects of microglia on neurogenesis following injury [319,328].

### 7.4. Adipokines

Leptin is predominantly produced by adipocytes, and its serum levels increase significantly in obesity, which is associated with development of leptin resistance [329,330]. It reaches the brain via direct transport through circumventricular organs, transport through the BBB and uptake into the brain parenchyma and choroid plexus [329,330]. In the hypothalamus, it acts in the central melanocortin system, i.e., the pro-opiomelanocortin (POMC) expressing neurons and the neurons producing agouti-related protein (AgRP), regulating hepatic glucose homeostasis, food intake and energy expenditure [329,330,331]. However, it also contributes to neurogenesis, synaptogenesis and neuronal function [332]. Besides the hypothalamus, leptin receptors are also expressed in the cortex, hippocampus, substantia nigra and other brain regions [332].

Microglial cells express long (active) and short isoforms of leptin receptors [207,332,333]. Leptin modulates the proinflammatory activation of microglia. It increases IL-6 expression through a mechanism involving insulin receptor substrate 1 (IRS-1), AKT, NF-κB and p300 [208]. It also enhances LPS-induced TNF, IL-1β and macrophage inflammatory protein 2 (MIP2) production in microglia in vitro [209]. However, leptin was also shown to reduce microglial inflammation, increase neuroprotective gene expression and preserve myelin in a spinal cord injury model [210]. Moreover, mice with myeloid cell-specific leptin receptor deficiency display impaired microglial ramification and decreased numbers of hypothalamic neurons, suggesting that leptin signaling is critical for proper microglial activation in the hypothalamus [207]. Mice bearing myeloid cell-specific leptin receptor deficiency are hyperphagic and display increased body weight and fat mass, underscoring the important role of microglial function in hypothalamic neuroendocrine responses [207].

Adiponectin is an adipokine with anti-inflammatory and insulin-sensitizing properties [334]. Its serum levels decrease with obesity, which contributes to development of chronic inflammation and insulin resistance [335]. Low-molecular-weight adiponectin oligomers are detected in the cerebrospinal fluid, and adiponectin receptors are abundantly expressed in the hippocampus and brain cortex [336,337,338,339]. Systemically administered adiponectin restrains microglia-mediated inflammation in the hypothalamus during short-term HFD feeding [340]. In accordance, aged adiponectin-deficient mice develop neuroinflammation, Aβ deposition, neuronal loss, central insulin resistance and memory deficits [341]. In addition, adiponectin knockout mice crossed with 5xFAD mice present accelerated amyloid deposition and reduced insulin sensitivity [342]. Moreover, adiponectin suppresses neuroinflammation in mice with corticosterone-induced depression [343,344]. In accordance, globular adiponectin reduces in vivo and in vitro LPS-induced microglial inflammation through an NF-κB-dependent mechanism [345]. Adiponectin also restrains Aβ-induced inflammatory activation and promotes an anti-inflammatory signature in microglia [346,347]. Moreover, adiponectin receptor agonists reduce microglial and astrocyte activation, and restore microglial Aβ phagocytοsis in AD and intracerebral hemorrhage mouse models, while adiponectin receptor suppression increases amyloidogenesis [342,348,349,350]. However, whether reduced adiponectin serum levels can account for obesity-associated neuroinflammation remains to be clarified.

### 7.5. Dyslipidemia

Obesity is associated with alterations in the serum lipidome (dyslipidemia) [351]. Circulating fatty acids are increased in obesity as a result of the enlarged adipose tissue and resistance to the antilipolytic effect of insulin [352,353]. Saturated fatty acids, such as palmitic and stearic acids, trigger inflammatory responses in a number of different cell types, such as monocytes/macrophages, endothelial cells, adipocytes, fibroblasts and smooth and skeletal muscle cells [352]. In humans, a diet rich in palmitate increases plasma IL-6 and IL-1β levels and potentiates proinflammatory effects of peripheral blood mononuclear cells [354]. Additionally, HFD-fed mice display increased serum levels of saturated fatty acids [355,356]. Circulating free fatty acids are taken up by the brain and their uptake is higher in obese compared to non-obese individuals [215,357,358]. For instance, brain uptake and accumulation of palmitate was shown in patients with obesity and metabolic syndrome [357]. Moreover, palmitate levels are increased in the cerebrospinal fluid of overweight and obese subjects with amnestic mild cognitive impairment [359]. In accordance, palmitate levels are elevated in the hypothalamus and hippocampus of HFD-fed mice, and their accumulation can be more pronounced in male than female mice [196,263,277,358,360,361].

Palmitate inhibits leptin signaling in a TLR-Myeloid differentiation primary response 88 (Myd88)-dependent manner [278]. In accordance, TLR4 loss-of-function mutation or intracerebroventricular injection of a TLR4 neutralizing antibody reduces obesity and leptin resistance [212]. Long-chain saturated fatty acids induce in the hypothalamus microglial inflammatory activation, while microglial depletion restores saturated fatty acid-induced leptin signaling impairment [268]. Intracerebroventricular administration of palmitate induces microglial activation and impairs synaptic plasticity in the mouse hippocampus, thereby leading to memory deficits [359]. In vitro, palmitate-treated microglia exhibit increased inflammatory gene expression and impaired migration and phagocytosis [196,211,213]. Mechanistically, saturated fatty acids induce TLR4 activation and downstream JNK signaling [212,214]. Moreover, they induce ER stress by increasing the expression of proteins of the unfolded protein response [277,362]. Palmitate also promotes inflammation through the reduction of peroxisome proliferator-activated receptor gamma coactivator 1-alpha (PGC1A) and estrogen receptor α (ERα) expression [361]. Furthermore, palmitate induces de novo ceramide synthesis in microglia, which in turn promotes inflammasome assembly and IL-1β secretion [200]. Ceramide also promotes TLR4 stabilization and thereby enhances LPS-induced inflammatory signaling [363]. The effects of saturated fatty acids on CNS inflammation may be reversed by unsaturated fatty acids, such as linoleic acid [356].

The brain is particularly enriched with the omega-6 polyunsaturated fatty acid (PUFA) arachidonic acid and the omega-3 PUFA docosahexaenoic acid [215,216]. PUFAs are present in the circulation as free fatty acids or as part of lipoproteins esterified to triacylglycerides, phospholipids or cholesteryl esters [216]. They are taken up in the brain through mechanisms, which are little understood [215,216]. Once in the brain, PUFAs are metabolized into downstream mediators or incorporated into membrane phospholipids, from which they can be later released [215,364]. PUFAs and their derivatives regulate cell survival, synaptic function, phagocytosis and microglia-mediated inflammation [215,216]. In general, an imbalance between omega-6 and omega-3 fatty acids resulting from western diet consumption may lead to chronic peripheral and central inflammation [216,365,366]. Many studies have demonstrated that omega-3 PUFAs down-regulate microglial inflammatory activation while promoting phagocytosis and resolution of inflammation [216,366]. In humans, higher omega-3 PUFA consumption is associated with lower risk of neurodegenerative disorders [215,216]. In accordance, western diet feeding increases, while a diet rich in docosahexaenoic acid decreases, amyloid deposition and microglial activation in the hippocampus of AD transgenic mice [217,218]. Moreover, omega-3 PUFAs enhance Aβ_42_ phagocytosis and decrease inflammation in human microglia [367]. Docosahexaenoic acid reduces surface presentation of TLR4 and CD14, thereby inhibiting LPS-induced downstream signaling and associated proinflammatory responses [219,220]. Additionally, dietary docosapentaenoic acid restrains microglia-mediated neuroinflammation and promotes neuronal survival in AD mouse models [368]. In accordance, omega-3 PUFA supplementation reduces microglial inflammatory activation and mitigates neural tissue destruction after traumatic brain injury [369,370]. In addition, docosahexaenoic and eicosapentaenoic acids reduce IFNγ- and myelin-induced microglial inflammatory activation, shift microglia to an anti-inflammatory phenotype and promote myelin phagocytosis in the cuprizone-induced demyelination model [371]. In conclusion, in the context of obesity an altered circulating lipidome can shape microglial function. In general, saturated fatty acids and omega-6 PUFAs favor neuroinflammation, while omega-3 PUFAs have anti-inflammatory and pre-resolving properties.

### 7.6. Insulin Resistance

Obesity is associated with hyperinsulinemia and development of peripheral and central insulin resistance [10,180,372,373]. In turn, central insulin resistance contributes to dysregulation of peripheral glucose and fat metabolism, thereby accelerating adiposity and associated metabolic disorders [372,374]. Epidemiological studies have shown that type 2 diabetes mellitus significantly increases the risk for cognitive decline or AD development [116,163,180,375]. Moreover, a clinical trial showed that moderate hyperinsulinemia induced by insulin infusion triggers an increase in cytokine and Aβ_42_ levels in the cerebrospinal fluid of healthy subjects [376]. In accordance, type 2 diabetes mellitus in overweight and obese individuals is linked to reduced neuronal viability [373].

Obesity-associated insulin resistance promotes amyloid deposition in the Tg2576 AD mouse model [377]. In accordance, mice generated by crossing APP23 mice with diabetic *ob/ob* or NSY mice display cerebrovascular inflammation and cognitive disturbances [378]. Furthermore, *db/db* mice display microgliosis, increased expression of proinflammatory cytokines and decreased BDNF hippocampal expression compared to control mice [236,379,380]. Mouse models of type 2 diabetes mellitus also demonstrate enhanced accumulation of α-synuclein and enhanced neuroinflammation when challenged with 1-methyl-4-phenyl-1,2,3,6-tetrahydropyridine (MPTP), a neurotoxin used to mimic cell death caused by PD [381]. In support of these findings, intracerebroventricular injection of insulin increases microglial inflammatory activation and Cyclooxygenase-2 (COX-2)/IL-1β levels in the hippocampus of mice [382]. The effects of insulin in the brain may become even more important in presence of peripheral inflammation since the latter enhances insulin transport into the brain [96]. Finally, macrophages chronically exposed to insulin can become insulin-resistant and display altered inflammatory responses [383]. Whether this holds true in microglia remains to be elucidated.

### 7.7. Glucocorticoids

Glucocorticoid levels may rise in obesity due to HPA axis activation and alterations in adrenocortical function; in turn, increased glucocorticoid levels promote abdominal fat deposition and insulin resistance [191,224,384,385,386]. HFD-fed, *db/db* and streptozotocin-treated mice all display increased corticosterone levels [191,224,225,226,227]. Chronically elevated corticosterone levels negatively affect CNS function leading to reduced brain volume, changes in grey and white matter structure, impaired hippocampal neurogenesis, reduced synaptic plasticity, memory deficits and depression-like behavior [201,283,284,387,388,389]. Although glucocorticoids exert potent anti-inflammatory effects [390], if chronically elevated, they may promote microglia-mediated neuroinflammation [192,193,391,392,393]. Microglia express glucocorticoid (GR) and mineralcorticoid (MR) receptors, and microglial function is regulated by glucocorticoids [394,395,396,397]. Chronic stress driving high corticosterone production leads to increased expression of microglial inflammatory markers, such as TNF, IL-6, IL-1β, iNOS, NF-κB and NLRP3, decreased expression of anti-inflammatory markers, including IL-1ra, IL-10, TGF-β and Ym1 (chitinase-like protein 3 (Chil3)), oxidative stress, and an enhanced inflammatory response to LPS in the rodent cortex and hippocampus [193,201,392,397,398]. Dexamethasone, a GR agonist, reduces dendritic spine density and induces proliferation and activation of microglia in the hippocampus of 3xTg-AD mice, while the GR antagonist mifepristone exerts opposite effects [399]. Moreover, stress in early life has lasting effects on microglia-mediated neuroinflammation and may aggravate AD pathology [400,401]. Pharmacological inhibition of corticosterone synthesis with metyrapone restrains microgliosis and decreases TNF and IL-1β expression in the hippocampus of *db/db* mice [402]. Moreover, inhibition of corticosterone action with mifepristone dampens LPS-induced proinflammatory responses and NLRP3 expression in the hippocampus in HFD-fed animals [227]. In addition, adrenalectomy and corticosterone replacement, pharmacological inhibition of glucocorticoid synthesis or lentiviral-mediated GR knockdown in *db/db* mice preserve hippocampal plasticity and learning function by restoring BDNF and TRKB expression in the hippocampus [225,403,404]. These studies collectively suggest that chronic exposure to glucocorticoids may prime microglia leading to enhanced inflammatory responses [396,405,406,407]. However, mice bearing GR deficiency in macrophages and microglia exhibit exacerbated neuronal damage after intraparenhcymal LPS injection [395]. GR-deficient microglia also display reduced motility, amoeboid morphology and increased proinflammatory activation in a PD animal model or in response to LPS [228,229,230,395]. These discrepancies could be due to brain region-, dose- or time-dependent differences in glucocorticoid effects [408,409,410], as well as brain region-dependent heterogeneity in microglial subpopulations [28].

### 7.8. The Gut-Brain Axis

Diet-induced obesity is associated with profound changes in the composition and function of the gut microbiome, termed dysbiosis [411,412]. Individuals with a low bacterial richness display more pronounced adiposity, systemic inflammation, insulin resistance and dyslipidemia compared to individuals with high bacterial richness [412]. Diet-induced alterations in the intestinal microbiome contribute to metabolic dysregulation and chronic inflammation in obesity [221,411,413,414]. Germ-free rodents are reported to be resistant to diet-induced obesity and associated insulin resistance [189,415,416] but display perturbed neurogenesis, myelination, BBB function, behavior and cognition [417]. Moreover, obesity is associated with enhanced gut permeability, leading to increased amounts of microbes and immunogenic bacterial products in the circulation [189,221,418]. Thus, microbiota can affect the host innate immune system, including microglia via microbial cell components, metabolites and endotoxins [189,190,221]. The intestinal microbiome composition was shown to be decisive for microglial cell proportions, maturation and innate immune responses [190,223]. Antibiotic treatment of mice with diet-induced obesity improves brain insulin signaling, neuroinflammation and depressive-like behavior, effects which are transferable to germ-free animals by fecal transplantation [222]. The effects of gut microbiota on microglia are also mediated by short-chain fatty acids deriving from bacterial fermentation and acting on G-protein coupled receptor 43 (GPR43) [190,223]. Microbiota-accessible carbohydrates, which prevent dysbiosis and decrease systemic inflammation, suppress microglial inflammatory activation and improve insulin signaling in the hippocampus of obese mice [419]. Roux-en-Y gastric bypass in obese rats leads to beneficial changes in gut microbiota accompanied by improved intestinal integrity; in addition, it improves microglia-mediated hypothalamic inflammation and energy intake control in a gut microbiome-dependent manner [420]. Moreover, probiotic treatment attenuates sickness behavior, microglial inflammatory activation, cerebral monocyte infiltration and TNF serum levels in mice suffering peripheral inflammation [421]. In contrast, a fiber-deficient HFD leads to microbiome alterations, gut barrier impairment and increased hippocampal microglial inflammation, effects that can be reversed by long-term supplementation with β-glucan [422]. To conclude, alterations in the gut microbiome can critically influence microglia-mediated neuroinflammation and even contribute to the pathogenesis of neurodegenerative diseases [417]. Several neurologic disorders, including AD, PD, MS and major depressive disorder, are associated with altered intestinal microbiota [417,423,424,425]. Hence, obesity-associated dysbiosis may drive disturbed microglial function and development of neurodegenerative conditions.

## 8. Conclusions and Future Perspectives

The effects of obesity are not limited to the periphery but also strongly affect neuronal and innate immune functions in the brain. Circulating immune cells, proinflammatory cytokines, adipokines, dyslipidemia, glucocorticoids and bacterial endotoxins are some of the factors generating a systemic environment unfavorable for microglial homeostatic function. The chronicity of such immune, metabolic and endocrine disturbances can have detrimental effects, as it may drive inflammatory priming, reduce microglial homeostatic functions and eventually exhaust microglia. In the hypothalamus, this can lead to derailment of whole body metabolism and acceleration of metabolic disease, and in the hippocampus or other brain regions to cognitive and mood disorders.

Although the link between metabolic disease and neurodegeneration is well supported by clinical data, less is known on the underlying mechanisms. Recent studies showed that in neurodegenerative disease microglia undergo reprograming from a homeostatic to a disease-associated state [76,78]. However, the mechanistic cues driving this transformation remain largely obscure. Moreover, it is not known whether a similar microglial shift also occurs in response to obesity or whether obesity may accelerate such microglial reprograming in response to proinflammatory or neurodegenerative stimuli.

A pallet of single-cell approaches is available today to dissect such phenomena. These technologies can overcome the discrepancies stemming from the heterogeneity between or within microglial or other myeloid cell populations [24,25,26,27,28]. Moreover, development of the *CX3CR1^Cre-ERT2^*, *P2ry12^Cre-ERT2^* and *Tmem119^Cre-ERT2^* mouse lines facilitate inducible microglia-specific gene deletion [426,427,428]. However, functional analyses are still hampered by the lack of appropriate in vitro microglia cell culture systems reliably reflecting the in vivo microglial properties. Although in vitro culture conditions can be improved through the addition of TGF-β2, IL-34 and cholesterol [429], the homeostatic profile of microglia is severely affected by in vitro culture [24,103]. Instead, ex vivo culture of brain slices more successfully preserves the physiological profile of microglia [430].

Aberrant microglial inflammation is a hallmark of several neurodegenerative disease. However, current knowledge on how to precisely and efficiently target microglia is limited. Complete depletion of microglia through genetic or pharmacological manipulations is possible and was shown to successfully ameliorate the course of neurodegenerative disease by reducing neuroinflammation in several animal studies [431]. For instance, microglia and macrophage removal through CSF1R inhibition improves EAE and cuprizone-induced demyelination [432,433] and prevents cognitive deficits in a model of cranial irradiation [434]. Moreover, in combination with environmental enrichment it reduces adipose tissue and hypothalamic inflammation and improves metabolic outcomes [435]. However, microglia elimination does not prevent or may even exacerbate amyloid pathology in AD mouse models [436,437] and aggravates brain ischemia, PD or coronavirus encephalitis [50,438,439]. Detrimental outcomes of microglia elimination in neurodegenerative disease are indicative for the critical role of microglia in resolution of inflammation and regeneration. After depletion, microglial populations are restored through self-renewal [431,440]. Enforced immune repopulation can reset a dysfunctional niche, as shown for Kupffer cells in the liver [441]. Similarly, repopulated microglia may acquire a preresolving function and promote brain tissue recovery [442].

Targeting microglia is challenging due to restriction of drug delivery by the BBB. In this aspect, small lipophilic molecules are promising candidates for microglia targeting. Steroid molecules with neuroprotective and anti-inflammatory properties, such as dehydroepiandrosterone (DHEA) or its derivatives, are especially attractive candidates [13,14,443,444,445]. Moreover, nanoparticles offer key advantages for drug delivery across the BBB [446]. Microglia targeting can be achieved through incorporation of ligands specific for microglial receptors on the nanoparticle structure [446]. In contrast, targeting of systemic factors that mediate peripheral and central inflammation may be less challenging. However, very few peripheral factors mediating microglia-mediated neuroinflammation have been studied in the context of obesity per se. Such studies are important since they could lead to the identification of targets for the management of neuroinflammation in metabolic disease.

Antidiabetic drugs, such thiazolidinediones and metformin, can restrain inflammation, while reduction of hyperglycemia may attenuate monocytosis [10,297]. A large meta-analysis showed that anti-inflammatory treatment effectively reduces symptoms of depression [447]. Furthermore, infliximab, a monoclonal antibody against TNF, exerts antidepressant effects only in patients with higher systemic inflammation and greater BMI [448], the latter pointing to the need of personalized treatments. Moreover, gender differences in microglial responses and occurrence of neurodegenerative diseases should be considered in the context of personalized therapy [80,449]. Finally, due to their applicability, dietary interventions offer attractive possibilities for the prevention of obesity-associated CNS inflammation. Return to a low-fat diet dampens neuroinflammation and restores hippocampal synaptic plasticity and cognition, although it only partially reverses adiposity [194]. Moreover, physical exercise efficiently combats obesity-associated chronic inflammation and may prevent neurodegenerative conditions [10,450,451]. Such interventions are safe and may be beneficial for both central and peripheral chronic immunometabolic disorders.

## Figures and Tables

**Table 1:** Factors that are discussed as mediators of the effects of obesity on microglia-mediated neuroinflammation.

Factor	Cause or Source	Effect on Microglia	References
TNF	Adipose tissue macrophages, adipocytes	Inflammation, reduced phagocytosis	[10,52,92,147,205,206]
Leptin	Adipocytes	Pro- and anti-inflammatory effects, ramification	[10,147,207,208,209,210]
Saturated fatty acids	Diet, adipose tissue	Inflammation, inflammasome activation, migration and phagocytosis	[196,200,211,212,213,214]
Omega-6/omega-3 fatty acids	Diet	Increased inflammation, decreased phagocytosis and reduced resolution of inflammation	[215,216,217,218,219,220]
Endotoxins	Gut microbiome	Inflammation	[221,222]
SCFA	Gut microbiome	Proper microglial function and homeostasis	[190,223]
Glucocorticoids	Adrenal glands	Anti- and pro-inflammatory effects	[191,192,193,224,225,226,227,228,229,230]
